# Perceptions of quality of life and the clinical learning environment: a cross-sectional study of four cohorts of health professions students

**DOI:** 10.1186/s12909-025-07894-1

**Published:** 2025-09-10

**Authors:** Per Östberg, Malin Sellberg, Per J. Palmgren, Riitta Möller

**Affiliations:** 1 Medical Unit Health Professions, Karolinska University Hospital, Stockholm, Sweden; 2 Department of Clinical Science, Technology and Intervention (CLINTEC), Division of Speech and Language Pathology, Karolinska Institutet, Stockholm, 171 77 Sweden; 3 Department of Neurobiology, Care Sciences and Society, Karolinska Institutet, Stockholm, 171 77 Sweden; 4 Department of Learning Informatics, Management and Ethics, Karolinska Institutet, Stockholm, 171 77 Sweden; 5 Department of Medical Epidemiology and Biostatistics, Karolinska Institutet, Stockholm, 171 77 Sweden; 6 Department of Clinical Science, Intervention and Technology, Karolinska Institutet, Stockholm, 171 77 Sweden

**Keywords:** Quality of life, Short form-12, Brunnsviken brief quality of life scale, Clinical learning environment, Undergraduate clinical education and environment measure

## Abstract

**Background:**

Health professions students may encounter a range of stressors during their clinical education that may impact their quality of life. This study aimed to explore how various health professions students perceive their quality of life and the environment in which they develop their clinical skills.

**Methods:**

An online survey was administered among registered undergraduate students in the physiotherapy, speech-language pathology, nursing, or medical programs. Quality of life was assessed using the Brunnsviken Brief Quality of Life Scale (BBQ) and the Short Form 12 (SF-12) questionnaire, which comprises Physical Component Summary (PCS) and Mental Component Summary (MCS) Scores. The results were compared to the outcomes of the Undergraduate Clinical Education and Environment Measure (UCEEM), which assesses students’ perceptions of the clinical learning environment.

**Results:**

In total, 280 students (median age 26 years, range 20–52; 72% females) responded. The mean total BBQ score for all students was 54.0 (SD 17.2), with no significant differences across the four programs. The mean MCS and PCS scores for all participants were 39.5 (SD 12.0) and 51.8 (SD 7.5), respectively, with significantly higher PCS scores among medical students than nursing students (*p* = 0.04). While the BBQ total score was weakly predicted by the UCEEM total score in the entire sample (ß = 0.19, 95% CI [0.06, 0.29], *p* = 0.002, *r*^2^ = 0.03), this prediction was substantially stronger in the speech-language pathology program (ß = 0.48, 95% CI [0.15, 0.87], *p* = 0.007, *r*^2^ = 0.23). Further, the UCEEM total score did not predict either the PCS or MCS scores in the entire study cohort.

**Conclusions:**

Our results do not support earlier findings that medical students have a much lower quality of life than other health professions students, although our medical students did rate their clinical learning environment lower than than the students in other programs. Nevertheless, our study raises concerns about the mental components of student’s quality of life. Therefore, educational institutions should seek support, both to students and supervisors in the clinical learning environment, so that possible problematic areas affecting students’ well-being and learning may be identified and addressed.

**Supplementary Information:**

The online version contains supplementary material available at 10.1186/s12909-025-07894-1.

## Introduction

The World Health Organization (WHO) defined quality of life (QoL) as individuals’ perception of their position in life, the context of the culture and value systems in which they live, and in relation to their goals, expectations, standards, and concerns [1, 2]. The concepts of QoL and health-related QoL (HRQoL) are sometimes used interchangeably. However, QoL is a broader concept that covers all aspects of life, while HRQoL, which consists of at least the domains of physical, psychological, and social functioning, focuses on the impact of possible illness and/or treatment on a person’s ability to live a fulfilling life [3]. In this paper, we employ the more extensive concept of QoL, which encompasses multiple characteristics, including physical well-being, psychological health, social relationships, and environmental conditions [4]. Although healthcare professionals are educated to attend to these aspects among their patients, their own QoL during training has received less attention [5].

Health professions students are subjected to considerable levels of physical and psychosocial stress, which could impact their QoL [6–10]. Potential stressors include academic workload, volume of information, competition with peers, examinations, and fear of failure [6, 11, 12]. However, there are a range of stressors operating at the various stages of education [13]. An important stage for students is the transition from campus-based studies to clinical training, where they are exposed to stressors such as lack of practical skills, stressed atmospheres among clinical staff, negative attitudes from staff and/or supervisors, or patient-related stressors such as human suffering and death [6, 12, 14]. Indeed, in several studies, medical students have been found to have lower perceived QoL than the general population, even when matched by age and gender [5, 13, 15, 16].

Studies on nursing students’ QoL have shown some divergent results. Cruz et al. [17] conducted a cross-sectional study among nursing students from nine countries. Overall, the students reported high scores for the physical domain of QoL and the lowest scores for the social domain. Labrague with colleagues [18] found that nursing students from three countries reported overall high scores for the social domain and lower scores for the physical dimensions of QoL, contradicting the results of the study of Cruz et al. [17] as well as those of Yildirim et al. [19], where students reported high physical dimension scores.

A few reports have included other health professions students in studies of QoL or conducted QoL comparisons between students from various study programs. Almhdawi et al. [6] studied HRQoL among more than 800 students from nine undergraduate programs, including physiotherapy and speech and audiology students. They found that the students had relatively low levels of physical and mental HRQoL and that there were no considerable differences among the students from different programs. In a study by Sklempe Kokic and colleagues [20], physiotherapy students were found to record lower QoL ratings than the national norm in most areas of HRQoL. Another study reported that speech-language pathology students had a predominantly good QoL but that there was a decrease in health satisfaction as the semesters advanced [21]. Taken together, these studies highlight the need to further examine students’ QoL.

Health professions students learn in diverse contexts, often referred to as learning environments [22, 23]. Clinical learning environments (CLEs) may be defined as any area where students apply theory to practice, where they conduct patient care to learn skills, attitudes, and decision-making abilities to become competent entry-level healthcare providers [24].

Few studies of health professions students have investigated the relationship between self-reported QoL and perceptions of the learning environments. A large Brazilian study comprising 1,350 medical students from 22 universities showed that there was a relationship between the students’ evaluations of their learning environment and QoL, regardless of how many years they had studied [25]. Another study by Tempski and colleagues [26] investigated the association between resilience, QoL, and perceived learning environment among medical students. They reported that students with a better QoL reported a better perception of their learning environment.

Thus, based on previous empirical findings, health professions students report subpar perceptions of their QoL. There is also a scarcity of research comparing and contrasting associations and differences between various health professions students’ perceptions of their QoL and clinical environment. Thus, the aim of the present study was twofold: [1] to assess QoL in students from four undergraduate programs, and [2] to gauge the relationship between students’ self-rated QoL and their perceprions of the CLE. We hypothesized that a more negative perception of the CLE would be associated with lower QoL scores.

## Methods

### Study design

This cross-sectional questionnaire study is part of a larger prospective research project examining students’ CLE, QoL, and interprofessional learning [27]. The Strengthening the Reporting of Observational Studies in Epidemiology (STROBE) statement for cross-sectional studies was used to ensure comprehensive reporting [28] (Additional file 1).

### Setting

The study was conducted in the context of four undergraduate study programs at a medical university, Karolinska Institutet (KI), in Stockholm, Sweden. The medical program is 5.5 years long, with the first two years covering preclinical education, and the remaining 3.5 years mainly covering clinical education. Clinical education makes up approximately 55% of the program, with placements lasting one to three weeks. Physiotherapy is a three-year program, with clinical education making up approximately 33% of the educational content and placements lasting one to eight weeks. Speech-language pathology is a four-year program, with clinical education making up approximately 20% of the educational content and placements lasting one to three weeks. Finally, nursing is a three-year program, with clinical education making up approximately 50% of the educational content and placements lasting five to six weeks. For all four programs, clinical training is organized at the university hospital, other hospitals around the university area, or at healthcare centers surrounding these hospitals in Stockholm, Sweden.

### Participants

A convenience sampling strategy was employed to recruit participants from the university’s major health science programs. To ensure a broad representation, students were invited from both shorter and longer programs as well as from earlier and later semesters. This approach enabled comparisons across Groups to explore how quality of life may change throughout the academic trajectory. Accordingly, invitations were extended to medical students in the sixth and 10th semesters, nursing and physiotherapy students in the third and sixth semesters, and speech-language pathology students in the fourth and sixth semesters. All the invited students were engaged in clinical training during the semester in which they were recruited. In total, 735 students registered for the semesters between April and November 2018 were eligible to participate. The students received oral information about the study, which was followed up by an e-mail containing a Link to the questionnaire and an attached information letter about the aims of the study. The letter also stated that participation was voluntary and that declining participation would not affect their education. By answering the questionnaire, the students Gave their consent to participate. In cases of no reply, four reminders were sent between four and 10 weeks after the initial dissemination. In total, 280 students (median age 26 years, SD 7.0, range 20–52; 72% females) returned the questionnaire, corresponding to a response rate of 38%. Among the respondents were 128 medical students, 72 nursing students, 49 physiotherapy students, and 31 speech-language pathology students. A majority of the students (191 or 68%) had previous working experience, while 160 (57%) had previous experience of university studies, and 59 (22%) had children. There were no differences between the participants and the nonparticipating students from the same semesters in teams of gender and age (data not shown). However, information regarding the number of students with children across different programs is currently unavailable.

### Data collection

The data were retrieved using an online questionnaire developed specifically for this study (Additional file 2). The instrument contained four sections: (1) sociodemographic data; (2) the Undergraduate Clinical Education Environment Measure (UCEEM) for assessing the CLE construct; along with (3) the Short Form 12 version 1 (SF-12 v1); and (4) the Brunnsviken Brief Quality of Life Scale (BBQ) for assessing the QoL construct. The socio-demographic data covered information regarding gender, age, number of children, educational background (previous university studies), and previous working life experience. Approximately 10–15 min were required to complete the questionnaire. Each participant received a book voucher as compensation.

#### The Brunnsviken brief quality of life scale (BBQ)

The BBQ is a brief QoL measure that assesses overall QoL using 12 items corresponding to satisfaction in six areas of life: leisure time, view on life, creativity, learning, friends and friendship, and view of self [29]. Each of these items is also scored in terms of satisfaction and importance on a Likert scale, where 0 corresponds to Strongly disagree and 4 to strongly agree. The satisfaction ratings are weighted by multiplying the ratings from the six areas of life by the satisfaction response to each life domain. The BBQ total score is calculated by multiplying the satisfaction and importance items for each life area and summing the six products to arrive at a total score (with a possible score range of 0 to 96). The higher the score, the better the QoL. The BBQ has demonstrated high validity, a good ability to differentiate between clinical and healthy groups, and sensitivity to change, making it suitable for screening and outcome measures. In a previous study, the mean score among a Swedish student sample consisting of undergraduate psychology students (mean age 26.8 years; SD 7.8) was 60.1 [29].

#### The short form 12 (SF-12 v 1)

The SF-12v1 is a Generic HRQoL questionnaire consisting of 12 questions measuring eight domains to assess physical and mental health [30]. This tool is not related to a specific age, disease, or population. The physical health-related domains are general health (GH), physical functioning (PF), role physical (RP), and bodily pain (BP). The mental health-related scales are mental health (MH), role emotional (RE), social functioning (SF), and vitality (V). The instrument has a recall period of four weeks. Each respondent’s SF-12 score is converted to two 0–100 scoring components: The Physical Component Summary Score (PCS) score and the Mental Component Summary (MCS) score (Quality Metric Health Outcome™) measure the latent concepts of physical and mental health, respectively [31]. Of the 12 items of the SF-12, 11 represent different aspects of health status, whereas the GH item is a question concerning overall health status. Studies have shown that the components of the SF-12 to be valid, reliable, and internally consistent. Higher PCS and MCS scores suggest a better HRQoL. The mean PCS and MCS scores (with standard deviations) among adults aged 18–74 years in Sweden are 50.3 (SD 8.5) and 52.9 (SD 9.6), respectively [32].

#### The UCEEM

The UCEEM is a tool used to investigate undergraduate students’ perceptions of the invitational, organizational, and pedagogical quality of the CLE in hospitals [33]. It was designed and validated for the Swedish context and has shown good psychometric properties [33]. The original UCEEM consists of 25 items scored on a 5-point Likert response scale, where 1 corresponds to fully disagree and 5 fully agree. The revised version comprises 26 items (personal correspondence with author Pia Strand, 18 September 2019), amounting to an overall score range of 26–130. The UCEEM has two overarching scales: experiential learning (score range 17–85) and social participation (score range 9–45). These scales represent fundamental elements of workplace learning, with experiential learning incorporating the construction of knowledge and meaning from real-life experience and social participation into participatory roles deemed appropriate for learners’ stage of training [34].

### Statistical analysis

The completed questionnaires were exported to Excel and imported into JASP, version 0.18.1. Cases with missing data were excluded listwise when calculating the total BBQ score, resulting in a total of 275 valid cases. The UCEEM total score was calculated based on 270 cases with complete data.

For descriptive purposes, the means, standard deviations (SD), and ranges were calculated for the quantitative variables. For the analysis of the categorical variables, the frequencies and relative frequencies (percentages) were calculated. Data distribution was assessed visually by contrasting possible discrepancies among the parameters of central tendency, evaluating the skewness of the distributions, and employing the Shapiro–Wilk test. The component summary scores for the SF-12 were derived from each participant’s responses through a computerized algorithm from Quality Metric. For comparisons of the differences between the groups, we used independent t-tests, with Cohen’s *d* reported as the effect size estimate, or analysis of variance (classical ANOVA or Welch’s ANOVA in case of unequal variances) with Omega-squared (ω^2^) used as effect size estimate. Significant main effects were further analyzed using Bonferroni post hoc analysis. Associations between the UCEEM scores and QoL measures (BBQ and SF-12 component scores) were assessed by simple Linear regression analysis, while the interrelationships between the QoL measures were estimated by Pearson correlation analysis. Probability values less than 0.05 were considered statistically significant for all the statistical tests.

## Results

### BBQ total scores

The mean BBQ total score for all students was 54.0 (SD 17.2; range 4–96) (Table 1). The physiotherapy and medical students had the highest mean BBQ scores (55.3 and 55.1, respectively). However, the BBQ total scores did not differ significantly across the four programs (ANOVA, *F* < 1, *p* = 0.49), nor was there a significant difference between the medical program on the one hand and the three shorter programs on the other (*t* = 1.04, *p* = 0.30).

There was no significant association between the BBQ total scores and age (*r* = −0.02, *p* = 0.69), nor was there any significant group difference in the mean BBQ scores between the older (> 26 years) (55.2; SD 16.3) and younger (53.2; SD 17.9) (*p* = 0.32) students. The mean total scores did not differ significantly across the demographic variables: female students (54.0; SD 17.1) compared to male students (53.8; SD 17.5); students with working experience (53.7; SD 17.1) versus those without (54.1; SD 17.2); students with) previous university study experience (52.9; SD 17.6 versus those without (55.4; SD 16.4); or students with children (57.1; SD 19.3) versus those without (53.8; SD 16.4).

**Table 1:** BBQ total, subscale, and item scores presented as means and standard deviations (SD), for all students and programs

		All students Mean (SD) *n* = 275	M students Mean (SD) *n* = 126	PT students Mean (SD) *n* = 48	SLP students Mean (SD) *n* = 31	*N* students Mean (SD) *n* = 70
Total score^a^		54.0 (17.2)	55.1 (16.7)	55.3 (18.4)	51.5 (19.1)	52.0 (16.4)
Item no						
Subscale 1. Leisure time		**7.5 (4.7)**	**7.7 (4.6)**	**8.7 (4.7)**	**6.9 (5.0)**	**6.7 (4.5)**
1	I am satisfied with my leisure time: I have the opportunity to do what I want in order to relax and enjoy myself.	2.0 (1.2)	2.1 (1.1)	2.3 (1.1)	1.9 (1.2)	1.8 (1.1)
2	My leisure time is important for my quality of life.	3.6 (0.7)	3.6 (0.7)	3.8 (0.4)	3.6 (0.7)	3.6 (0.7)
Subscale 2. View on life		**9.8 (4.2)**	**10.0 (4.2)**	**9.4 (4.1)**	**9.7 (5.3)**	**9.8 (3.9)**
3	I am satisfied with how I view my life: I know what means a lot to me, what I believe in, and what I want to do with my life.	2.9 (1.0)	3.0 (0.9)	2.7 (1.0)	2.8 (1.2)	2.9 (0.9)
4	How I view my life is important for my quality of life.	3.3 (0.7)	3.3 (0.8)	3.4 (0.6)	3.4 (0.7)	3.3 (0.6)
Subscale 3. Creativity		**6.5 (4.2)**	**6.3 (4.0)**	**6.9 (4.4)**	**6.5 (4.9)**	**6.7 (4.2)**
5	I am satisfied with opportunities to be creative: to get to use my imagination in my everyday life, in a hobby, on the job, or in my studies.	2.3 (1.1)	2.2 (1.0)	2.4 (1.1)	2.3 (1.2)	2.4 (1.0)
6	Being able to be creative is important for my quality of life	2.9 (1.0)	2.8 (1.0)	3.0 (1.0)	2.9 (1.3)	2.9 (1.0)
Subscale 4. Learning		**9.2 (4.3)**	**9.6 (4.3)**	**9.4 (4.1)**	**8.6 (4.4)**	**8.7 (4.3)**
7	I am satisfied with my learning: I have the opportunity and desire to learn new, exciting things and skills that interest me.	2.7 (1.0)	2.8 (1.0)	2.8 (0.9)	2.7 (1.0)	2.7 (1.0)
8	Learning is important for my quality of life.	3.3 (0.8)	3.4 (0.8)	3.3 (0.7)	3.2 (0.7)	3.2 (0.8)
Subscale 5. Friends and friendship		**10.8 (4.9)**	**11.0 (4.7)**	**11.5 (4.9)**	**10.3 (4.7)**	**10.0 (5.3)**
9	I am satisfied with friends and friendship: I have friends that I associate with and who support me (as many friends as I want and need).	2.9 (1.1)	3.0 (1.1)	3.1 (1.0)	2.9 (1.0)	2.8 (1.2)
10	Friends and friendship are important for my quality of life.	3.6 (0.7)	3.7 (0.7)	3.7 (0.6)	3.4 (0.8)	3.4 (0.8)
Subscale 6. View of self		**10.3 (4.7)**	**10.7 (4.4)**	**9.5 (4.7)**	**9.6 (5.2)**	**10.4 (4.8)**
11	I am satisfied with myself as a person: I like and respect myself.	2.8 (1.1)	2.9 (1.0)	2.6 (1.1)	2.7 (1.2)	2.9 (1.1)
12	My satisfaction with myself as a person is important for my quality of life.	3.6 (0.7)	3.6 (0.7)	3.6 (0.6)	3.6 (0.6)	3.5 (0.7)

*BBQ *Brunnsviken Brief Quality of life Scale, *n *number of students with complete data, *M *Medical, *PT P*hysiotherapy, *SLP* Speech-language pathology, *N* Nursing

^a﻿^The BBQ total score may range from 0 to 96

### SF-12 domains

#### MCS scores

The mean MCS score for all participants was 39.5 (SD 12.0) (Table 2). The mean MCS scores were relatively equal among students from the different programs, with no significant main effect of the program (*F*(3, 276) = 2.27, *p* = 0.08). There was no significant difference between the medical program and the three shorter programs (*t* = 1.37, *p* = 0.17).

**Table 2:** SF-12 summary components presented as means and standard deviations (SD) for all students and programs

	SF-12 summary components	
Participants	Mean MCS (SD)	Mean PCS (SD)
All students (*n* = 280)	39.5 (12.0)	51.8 (7.5)
Medical students (*n* = 128)	40.6 (12.0)	53.2 (6.7)
Physiotherapy students (*n* = 49)	39.1 (13.5)	51.5 (5.5)
Speech-language pathology students (*n* = 31)	37.9 (10.9)	51.9 (8.4)
Nursing students (*n* = 72)	38.6 (11.4)	49.5 (9.1)

*SF-12* The Short Form 12, *SD* Standard Deviation, *MSC* Mental Health Composite Scale, *PCS* Physical Composite Scale, *n* number of students

There was no significant correlation between age and the MCS scores (*r* = 0.02, *p* = 0.80). Moreover, upon dividing the participants into two cohorts with the mean age of 26 years serving as the cut-off, there was no significant difference in the mean MCS scores between the younger (up to 26 years) and older (over 26 years) students (40.3; SD 11.9 versus 39.2; SD 12.1, respectively) (*p* = 0.46).

The mean MCS scores showed no significant differences across demographic variables, including gender (male 41.0, SD 12.9; female 39.2, SD 11.6), previous university study experience (with 38.9, SD 12.0; without 40.4, SD 11.9), or parental status (with children 41.1, SD 11.4; without children 39.5, SD 12.1). However, students with previous working experience had a lower mean MCS score (38.4; SD 12.0) than those without working experience (42.0; SD 11.7) (Cohen’s *d* = 0.30, *p* = 0.02).

#### PCS scores

The mean PCS score for all participants was 51.8 (SD 7.5) (Table 2). A comparison across the programs showed that the medical students had the highest mean PCS score, followed by physiotherapy, speech-language pathology, and nursing students. There was a significant main effect of the program (Welch’s ANOVA, *F*(3, 93.942) = 2.77, *p* = 0.046; *ω*^2^ = 0.021), with a significant difference between medical and nursing students (*p* = 0.04).

Furthermore, there was no clear relationship between the PCS and age (*r* = −0.08, *p* = 0.20). However, as with the MCS scores, the male students had a slightly higher but not significantly different mean PCS score (mean 52.9; SD 6.1) than the female students (51.3; SD 8.0). The PCS scores did not show significant differences between students with (51.6; SD 7.7) and without (52.1; SD 7.1) previous working experience, those with (51.2; SD 7.7) and without (52.6; SD 7.2) previous study experience, or those with (51.8; SD 7.2) and without (51.9; SD 7.4) children.

### UCEEM total scores

Data on the UCEEM total, item, and subscale scores have been published previously [27], and therefore, only the main results are presented here. The overall mean UCEEM score for all students was 98.3 (SD 18.4). The physiotherapy students had the highest mean UCEEM total score (112.3; SD 12.9), followed by the speech-language pathology (108.4; SD 17.7), nursing (97.9; SD 20.3), and medical (90.1; SD 14.8) students. As previously reported [27], the physiotherapy and speech-language pathology students exhibited significantly higher UCEEM total scores than the medical students (*p* < 0.001 for both) and nursing (*p* < 0.001 and *p* < 0.01, respectively) students. However, the difference between the physiotherapy and speech-language pathology programs was not significant. Even the nursing students had significantly higher scores than the medical students (*p* < 0.01) [27].

### The relationship between the UCEEM and QoL measures

Simple linear regression analysis was used to assess whether the CLE perceptions indicated by the UCEEM total score were associated with any of the QoL measures. The BBQ total score was significantly but very weakly predicted by the UCEEM total score in the sample as a whole (ß = 0.19, 95% CI [0.06, 0.29], *p* = 0.002, *r*^2^ = 0.03). However, subgroup analyses indicated that the strength of the relationships between the UCEEM and QoL measures varied across programs. In the SLP program, the BBQ total score was substantially predicted by the UCEEM total score (ß = 0.48, 95% CI [0.15, 0.87], *p* = 0.007, *r*^2^ = 0.23), but the relationship was weaker in the medical program (ß = 0.26, 95% CI [0.10, 0.49], *p* = 0.003, *r*^2^ = 0.07) and very weak and nonsignificant in the nursing program (ß = 0.19, 95% CI [−0.05, 0.35], *p* = 0.13, *r*^2^ = 0.04) and the physiotherapy (ß = 0.04, 95% CI [−0.43, 0.55], *p* = 0.81, *r*^2^ = 0.001) programs.

Neither the PCS nor the MCS scores were predicted by the UCEEM total score (*p* = 0.18 and *p* = 0.08, respectively) in the entire cohort. Subgroup analyses showed no significant relationship between the PCS and UCEEM scores in any of the four programs. However, the MCS scores were significantly predicted by the UCEEM scores in the medical program (ß = 0.20, 95% CI [0.02, 0.30], *p* = 0.02, *r*^2^ = 0.04), but the UCEEM scores did not significantly predict the MCS scores in the other three programs.

Considering the interrelationships between the QoL measures, the BBQ total score correlated moderately with the MCS score (*r* = 0.54, *p* < 0.001) and weakly with the PCS score (*r* = 0.22, *p* < 0.001) in the sample as a whole.

## Discussion

The two main aspects of this study, which covered 280 students from four different programs, were QoL, measured by the BBQ and SF-12, and perceptions of the CLE. The results revealed that the BBQ scores did not differ significantly across the four programs; however, they were slightly lower than previously reported scores among psychology students [29]. Further, the students in the current study exhibited higher physical (PCS) but lower mental (MCS) QoL scores than in the normative data representing the Swedish population [32]. The BBQ total scores weakly predicted the UCEEM scores but were not significantly associated with the MCS or PCS scores in the sample as a whole, even if we found differences within the programs.

The BBQ was created as a brief, easy-to-use, and freely accessible self-rating QoL scale congruent with the conceptualization of overall life satisfaction [29]. The instrument is unifactorial and gauges six areas in life, accompanied by statements about whether or not they are important to a respondent. Thus, Lindner and colleagues [29] articulated that the BBQ probes whether the respondent is satisfied with the parts of life that matter most. In our sample of health professions students, the mean BBQ overall score was 54.0. Comparisons of our findings with those of other contexts are difficult because of the dearth of investigations that have employed the BBQ instrument in the student population, particularly health professions students. However, in a sample of psychology students (*n* = 163), the authors reported a mean BBQ score of 60.1, indicating that our student sample displayed a somewhat lower overall average, albeit with a rather similar data spread [29].

In the present study, no differences were detected between the overall BBQ score and the demographic variables, such as age or educational background. It is also rather encouraging that we did not discover any substantial gender differences. These findings are congruent with results described by other scholars [35, 36] who reported no, or only minor, gender differences, and potentially reflect the relative gender equality in Sweden and the Nordic countries. Although not significant, there was some indication that health professions students with children were characterized by higher overall life satisfaction compared to those without children. This trend is in concurrence with that presented by Cesar et al. [37], who assessed QoL among master’s and doctoral students in health and reported that having children was related to average psychological health. These authors postulated that this positive relationship may be associated with having children and can represent a sense of purpose and augmented subjective well-being. We detected various statistical mean differences on individual item levels in the BBQ instrument. However, the BBQ was designed to measure a single latent factor corresponding to overall subjective self-reported QoL [29]. Consequently, from an educational research perspective, any data interpretation of statistical differences on an item level can be misleading and redundant.

In the current study found that the health professions students showed higher physical (51.8) but lower mental (39.5) QoL scores, as measured by the SF-12, compared to normative data from the Swedish population (PCS 50.3 and MCS 52.9, respectively) [32]. Although the lower MCS scores in comparison to those of the general population were surprising, these results are consistent with QoL data reported from student populations in other contexts and countries [19, 38, 39]. Indeed, health professions students, and medical students in particular, have been found to have lower perceived QoL than the general population, even when matched by gender [5, 16]. It has been proposed that health professions students may face a multitude of stressors that may affect their sense of well-being and have the potential to affect their mental health and perceived QoL. These stressors may include factors such as lack of free time, extensive academic workload load, and fear of failure [19, 26, 40]. Nevertheless, we did not explore possible explanations for the students’ QoL and well-being.

CLEs play an important role in health professions students’ attainment of clinical competence for future practice [23, 24]. Enns et al. [25] found a consistently positive association between QoL measures and the CLE scores. Further, Temspki et al. [26] reported that medical students had a better QoL as well as better perceptions of their learning environment if they had higher resilience levels. While the current study did not employ an instrument to measure resilience, the medical students recorded the lowest ratings for their CLE, while their QoL, measured using the BBQ was among the highest. Further, the medical students had the highest physical QoL component score. A reasonable point of contention could be whether the SF-12 is a valid measure of QoL among a generally relatively healthy young population as in the case of higher education students.

However, subgroup analyses indicated that there were significant relations between the UCEEM and BBQ scores only in the SLP and medical programs, but not in the nursing and physiotherapy programs. SLP students rated lowest on the BBQ but the second highest on the UCEEM, concurrently exhibiting the strongest positive linear correlation between these measures among the programs. Furthermore, there was a weak relationship between the UCEEM and MCS scores specifically in the medical program. Explaining these results is challenging, perhaps due to several methodological, individual, and contextual factors in clinical training, suggesting that perceptions of the CLE interact with measures of life quality in a more complex way than implied by the overall ratings.

One strength of our study is that we included students from four different programs including earlier and later semesters, which may have decreased the fluctuations among the student cohorts. We also used two standardized and validated instruments to measure different QoL aspects that allowed comparisons between other study samples. Further, the gender distribution among the respondents corresponded to that of universities in Nordic countries in general. However, there are several potential limitations to our study. First, our study is cross-sectional, and causality cannot be assessed. Second, the data were collected through self-reports, which may have led to unreliable responses due to, for example, misinterpretations, thereby contributing to the risk of volunteer bias. Although this limitation is well known, self-reports are regularly used in research due to their low cost and efficiency. While this study compares QoL across different healthcare student groups, such comparisons should be interpreted with caution. The nature of training, clinical exposure, academic demands, and professional identity formation may vary among students across the studied programs. These differences may influence students’ perceptions of QoL, potentially confounding direct comparisons. Nevertheless, despite inherent differences, identifying patterns of QoL across student groups may inform the development of more equitable and responsive educational environments. Our choice of a second instrument, the SF-12, for assessing QoL might not have been optimal for gauging differences among the educational programs in a relatively healthy and relatively young population. In addition, despite the BBQ showing robust psychometric characteristics, it is still a relatively new instrument, thus limiting the scope of comparisons. Finally, as a result of the low response rate among nursing students, the results should be interpreted with caution. Nevertheless, we consider the sample to be of sufficient variety and size to provide valuable data.

As we conducted a cross-sectional study, further studies should explore students’ QoL patterns throughout their studies, especially during more stressful transitions, such as at the beginning of their studies or following the start of their clinical rotations. Such an approach could help identify factors, e.g., contributing to lower levels of mental health. Further, multicenter studies would be required for more diverse samples and to compare data from a range of faculties and organizations. Qualitative approaches, such as interviews, would also allow for a deeper analysis of the factors affecting students’ QoL.

## Conclusions

Our results do not support earlier conclusions showing that medical students have a much lower QoL than other health professions students, even though their CLE ratings were lower than those of other students. Nevertheless, our study raises concerns regarding the mental components of the student’s QoL. Consequently, educational institutions should seek to support, both students and supervisors in the CLE, so that possible problematic areas affecting students’ well-being and learning may be identified and addressed.

## Supplementary Information

Supplementary Material 1.

Supplementary Material 2.

## Data Availability

No datasets were generated or analysed during the current study.

## Abbreviations

BBQ: Brunnsviken Brief Quality of Life Scale
CLE: Clinical learning environment
CoP: Communities of Practice
ECTS: European Credit Transfer and Accumulations system
MCS: Mental Component Score
PCS: Physical Component Score
PT: Physiotherapy
SF-12: The Short Form 12
SLP: Speech-language pathology
UCEEM: Undergraduate Clinical Education Environment Measure
