# Supplementary material for: Perceptions of quality of life and the clinical learning environment: a cross-sectional study of four cohorts of health professions students
Source: BMC Med Educ. 2025 Sep 10;25:1252. doi: 10.1186/s12909-025-07894-1 (PMC12421748; doi:10.1186/s12909-025-07894-1)
Supplement: Supplementary file 2 — Supplementary Material 2. [file 12909_2025_7894_MOESM2_ESM.docx]

# Learning Environment and Quality of Life

## Questions about your education and educational background

**Which educational program are you currently enrolled in?**

**When did you start your educational program?**

**Which semester are you currently in?**

**Have you worked in another profession before starting this vocational education?**

If yes, please specify:

**Have you previously studied at a university or university college?**

If yes, please specify:

# Statements about the Clinical Learning Environment

Response options: ☐ 1 ☐ 2 ☐ 4 ☐ 4 ☐ 5

(1 = Strongly disagree, 5 = Strongly agree)

**I received useful induction to this placement.**

**My supervisors were expecting me when I arrived.**

**My (work) tasks are relevant to the learning objectives.**

**I am sufficiently occupied with meaningful (work) tasks.**

**My tasks are suitably challenging for my level of knowledge and skills.**

**I am encouraged to participate actively in the work here.**

**I have adequate access to computers.**

**There is sufficient physical space for the number of students on placement here.**

**I have a supervisor to whom I know I can turn.**

**I have sufficient access to supervision.**

**The supervisors are well prepared for supervising.**

**It is clear that my supervisors are familiar with the learning objectives.**

**I receive useful feedback from my supervisors.**

**I feel able to ask my supervisors any question I wish.**

**I get the opportunity to provide a rationale for my actions during supervision sessions.**

**My problem-solving skills are developing well in this placement.**

**I have the opportunity to put my theoretical knowledge into practice in this placement.**

**I have the opportunity to learn together with other students in this placement.**

**As a student I am received in a positive way by the staff here.**

**I feel included in the team of people who work here.**

**I feel welcome in the staff room/lunch room here.**

**Communication between those working here is good.**

**Everyone is treated equally here, regardless of cultural background.**

**Everyone is treated equally here, regardless of gender.**

**Everyone is treated equally here, regardless of professional background.**

**I feel I have influence over my learning in this placement.**

**Below, you are welcome to list other factors that you believe are important for the clinical learning environment, as well as suggestions for how it can be improved:**

# Questions about Health-Related Quality of Life

**In general, would you say your health is:**

☐ Excellent ☐ Very good ☐ Good ☐ Fair ☐ Poor

**Moderate activities, such as moving a table, vacuuming, hiking, or gardening:**

☐ Yes, limited a lot ☐ Yes, limited a little ☐ No, not limited at all

**Climbing several flights of stairs:**

☐ Yes, limited a lot ☐ Yes, limited a little ☐ No, not limited at all

**Accomplished less than you would like (due to physical health):**

☐ Yes ☐ No

**Were limited in the kind of work or other activities (due to physical health):**

☐ Yes ☐ No

**Accomplished less than you would like (due to emotional problems):**

☐ Yes ☐ No

**Did work or other activities less carefully than usual (due to emotional problems):**

☐ Yes ☐ No

**How much did pain interfere with your normal work:**

☐ Not at all ☐ A little bit ☐ Moderately ☐ Quite a bit ☐ Extremely

**Have you felt calm and peaceful?**

☐ All of the time ☐ Most of the time ☐ A good bit of the time ☐ Some of the time ☐ A little of the time ☐ None of the time

**Did you have a lot of energy?**

☐ All of the time ☐ Most of the time ☐ A good bit of the time ☐ Some of the time ☐ A little of the time ☐ None of the time

**Have you felt downhearted and blue?**

☐ All of the time ☐ Most of the time ☐ A good bit of the time ☐ Some of the time ☐ A little of the time ☐ None of the time

**How much of the time has your physical health or emotional problems interfered with your social activities?**

☐ All of the time ☐ Most of the time ☐ Some of the time ☐ A little of the time ☐ None of the time

# Questions about Quality of Life

Response options: ☐ 0 ☐ 1 ☐ 2 ☐ 3 ☐ 4

(0 = Strongly disagree, 4 = Strongly agree)

I am satisfied with my leisure time: I have the opportunity to do what I want in order to relax and enjoy myself.

My leisure time is important for my quality of life.

I am satisfied with how I view my life: I know what means a lot to me, what I believe in, and what I want to do with my life.

How I view my life is important for my quality of life.

I am satisfied with opportunities to be creative: to get to use my imagination in my everyday life, in a hobby, on the job, or in my studies.

Being able to be creative is important for my quality of life.

I am satisfied with my learning: I have the opportunity and desire to learn new, exciting things and skills that interest me.

Learning is important for my quality of life.

I am satisfied with friends and friendship: I have friends that I associate with and who support me (as many friends as I want and need).

Friends and friendship are important for my quality of life.

I am satisfied with myself as a person: I like and respect myself.

My satisfaction with myself as a person is important for my quality of life.

# Questions about You

**What gender do you identify with?**

☐ Woman ☐ Man ☐ Other ☐ Prefer not to say

**What is your age?**

**Do you have children?**

☐ Yes ☐ No ☐ Prefer not to say
